# Machine Learning Classification of Pediatric Health Status Based on Cardiorespiratory Signals with Causal and Information Domain Features Applied—An Exploratory Study

**DOI:** 10.3390/jcm13237353

**Published:** 2024-12-02

**Authors:** Maciej Rosoł, Jakub S. Gąsior, Kacper Korzeniewski, Jonasz Łaba, Robert Makuch, Bożena Werner, Marcel Młyńczak

**Affiliations:** 1 Institute of Metrology and Biomedical Engineering, Faculty of Mechatronics, Warsaw University of Technology, 02-525 Warsaw, Poland; kacper.korzeniewski.dokt@pw.edu.pl (K.K.); jonasz.laba.dokt@pw.edu.pl (J.Ł.); marcel.mlynczak@pw.edu.pl (M.M.); 2 Department of Pediatric Cardiology and General Pediatrics, Medical University of Warsaw, 02-091 Warsaw, Poland; jakub.gasior@wum.edu.pl (J.S.G.); bozena.werner@wum.edu.pl (B.W.); 3 Department of Physical Education, Kazimierz Pulaski University of Technology and Humanities in Radom, 26-600 Radom, Poland; r.makuch@uthrad.pl

**Keywords:** cardiorespiratory parameters, machine learning, causality, XAI, cardiorespiratory coupling, health status

## Abstract

**Background/Objectives:** This study aimed to evaluate the accuracy of machine learning (ML) techniques in classifying pediatric individuals—cardiological patients, healthy participants, and athletes—based on cardiorespiratory features from short-term static measurements. It also examined the impact of cardiorespiratory coupling (CRC)-related features (from causal and information domains) on the modeling accuracy to identify a preferred cardiorespiratory feature set that could be further explored for specialized tasks, such as monitoring training progress or diagnosing health conditions. **Methods:** We utilized six self-prepared datasets that comprised various subsets of cardiorespiratory parameters and applied several ML algorithms to classify subjects into three distinct groups. This research also leveraged explainable artificial intelligence (XAI) techniques to interpret model decisions and investigate feature importance. **Results:** The highest accuracy, over 89%, was obtained using the dataset that included most important demographic, cardiac, respiratory, and interrelated (causal and information) domain features. The dataset that comprised the most influential features but without demographic data yielded the second best accuracy, equal to 85%. Incorporation of the causal and information domain features significantly improved the classification accuracy. The use of XAI tools further highlighted the importance of these features with respect to each individual group. **Conclusions:** The integration of ML algorithms with a broad spectrum of cardiorespiratory features provided satisfactory efficiency in classifying pediatric individuals into groups according to their actual health status. This study underscored the potential of ML and XAI in advancing the analysis of cardiorespiratory signals and emphasized the importance of CRC-related features. The established set of features that appeared optimal for the classification of pediatric patients should be further explored for their potential in assessing individual progress through training or rehabilitation.

## 1. Introduction

The assessment of cardiovascular function in ambulatory or field conditions (e.g., during physical training, athletic monitoring, or routine primary care visits) has predominantly relied on electrocardiography (ECG), a non-invasive measurement of the electrical activity of the heart. The intervals between the consecutive R peaks from a QRS complex detected from ECG recordings can be used to calculate the heart rate variability (HRV) parameters in the time, frequency, and nonlinear domains, which constitute valuable markers in various health conditions [1,2,3,4]. Importantly, many studies emphasized the value of incorporating respiratory data, such as the respiratory rate (RespRate), tidal volume (TV), and pulmonary ventilation, to enhance the clinical relevance of HRV analysis [2,5,6,7]. Moreover, respiration acts as a confounder for cardiovascular and cerebrovascular controls [8] and is necessary for the assessment of the baroreflex role [9]. Recently, there has been a growing interest in introducing new cardiorespiratory parameters, which could benefit from the diagnostic information hidden in the interdependence and cooperation of cardiac and respiratory systems [10]. This linkage is known as cardiorespiratory coupling (CRC), which is reflected in phenomena like respiratory sinus arrhythmia (RSA) or baroreceptor coupling [11]. These interdependencies can be quantified based on the HRV associated with breathing [12] or by using parameters from the causal or information domains, which simultaneously utilize both cardiac and respiratory signals for such quantification [13,14,15,16]. The causal analysis of cardiorespiratory signals, mostly based on the Granger causality (GC), allows for the identification and quantification of directional influences between the cardiac and respiratory systems. By analyzing the temporal sequence of events, the GC can determine whether changes in one system can improve the prediction of changes in the other, providing insight into the interplay between both heart and lung functions. When testing the causal influence, e.g., from the respiratory signal to the tachogram (denotated as Resp→RR), with this method, two autoregressive models are created. The first model predicts the current value of the tachogram based on the p defined number of past values of this signal (Equation (1)), while the second model predicts the same current value of the tachogram but based on the past values of the cardiological and respiratory signals (Equation (2)):

(1) $$RR\left(t\right)=\sum_{i=1}^{p}A_{i}RR\left(t-i\right)+\varepsilon_{1}$$

(2) $$RR\left(t\right)=\sum_{i=1}^{p}B_{i}RR\left(t-i\right)+\sum_{i=1}^{p}C_{i}Resp\left(t-i\right)+\varepsilon_{2}$$

Then, the measure of the causality Resp→RR can be defined as the logarithm of the ratio of the variances of the models’ residuals $\varepsilon_{1}$ and $\varepsilon_{2}$, as shown in Equation (3) [17]:

(3) $${GC}_{Resp\to RR}=ln\;\frac{\sigma^{2}\varepsilon_{1}}{\sigma^{2}\varepsilon_{2}}$$

While traditional GC relies on linear modeling, more sophisticated nonlinear approaches were also developed to enable the analysis of more complex relationships [18,19,20]. The information domain quantification of the interdependencies between signals is mostly based on the entropy parameters [21,22]. Both causal- and information-based parameters are commonly applied to detect direct and indirect couplings in time series; thus, they are also useful for CRC quantification [14,23,24]. Notwithstanding, there is a lack of literature on the possible descriptive and diagnostic utility of such parameters.

As computational power increases and more cardiorespiratory parameters become available, the use of machine learning (ML) tools for biomedical data analysis becomes more popular [25]. This trend is advancing the fields of precision and individualized medicine [26,27]. ML algorithms and wearable devices play a crucial role in these contexts, enabling continuous monitoring of physiological signals and advanced analysis of data to support tailored interventions [28,29]. Personalized information about a subject’s health status, based on cardiorespiratory data, can be presented either as a continuous parameter (corresponding to a regression problem in ML) or as discrete labels (through ML classification). To achieve precise personalization, it is essential to identify the physiological parameters that most accurately reflect an individual’s health condition and enable differentiation between various health statuses. Determining these key parameters enables further tailoring of ML models for personalized insights, preferably based on data gathered from wearable devices. Such insights enable clinicians and coaches to customize interventions effectively and monitor progress with greater precision. Thus, determining the most relevant features from a broad range of cardiorespiratory data is a critical first step in enhancing diagnostic accuracy and improving individualized care. Despite ML models achieving human-level performance across various tasks, their perception as inscrutable “black boxes” greatly limits the understanding of their decision-making foundations, thus undermining their broader acceptance and application in medicine [30]. To address this issue, the use of explainable artificial intelligence (XAI) techniques has gained popularity. These methodologies play a crucial role in enhancing the interpretability and trustworthiness of ML models, thereby elevating their utility within professional settings. This progress is crucial in bridging the gap between complex ML algorithms and real-world applications, ensuring that their integration into various domains is both effective and ethically responsible [31].

With the growing emphasis on personalized medicine, there is an increasing demand for individualized assessments of health status to optimize treatment, rehabilitation, workouts, and intervention strategies [32,33,34]. For instance, CRC was recently used to determine the optimal breathing training frequency [35]. Individualized approaches are particularly important in pediatric populations, where only 40% of youth are currently believed to have an optimal cardiorespiratory fitness (CRF) level, a crucial marker of physical and mental health, as well as academic achievement [36]. Furthermore, assessing one’s health status in terms of CRF and muscular fitness is essential for young individuals, as both are positively associated with health-related quality of life, particularly in the physical, psychological, and social domains in this population [37]. Moreover, higher CRF during childhood and adolescence is associated with better cardiometabolic health parameters later in life, emphasizing the long-term benefits of early interventions targeting CRF [38]. These factors highlight the importance of individualized assessments of health status in pediatric populations. In this study, we made an effort to explore the capabilities of ML in classifying the health statuses of pediatric subjects from three distinct groups. This allowed for the identification of an optimal set of cardiorespiratory features and lay the groundwork for further personalized modeling.

This study aimed to evaluate the accuracy of ML techniques in classifying pediatric individuals with respect to their health status—including patients with heart disease, healthy participants, and trained athletes—based on cardiorespiratory features calculated from short-term measurements taken under static conditions. Additionally, this study investigated the importance of CRC-related features by examining their influence on modeling accuracy, hypothesizing that these features capture unique physiological interactions between cardiac and respiratory systems, thereby introducing additional information about the subject’s health status and improving the performance of machine learning models. Moreover, this evaluation was performed to establish a preferred set of features that could be used for further development in more specialized classification or regression tasks related to assessing individual progress through training or rehabilitation or diagnosing specific health conditions.

## 2. Materials and Methods

### 2.1. Study Design

The inclusion criteria for this study were ages between 6 and 18 years old and given written informed consent, while the exclusion criteria were signs of infection and diagnosed additional disorders that may affect the functioning of the autonomic nervous system. Subjects were assigned to three distinct groups (which also served as labels for the ML classification) according to their health status based on the following criteria:

- Cardiac—subjects with an ongoing cardiac disease requiring hospitalization;
- Healthy—subjects without any active heart disease, whether sedentary or recreationally active subjects according to McKay classification [39];
- Sport—trained adolescent athletes [39,40] (soccer players) affiliated with a sports club, with at least 3 years of training experience and regularly training ∼3 times per week with a purpose to compete.

For the cardiorespiratory data acquisition, all participants took part in ECG and impedance pneumography (IP) recordings performed for at least 5 min at rest in the supine position using the Pneumonitor device. This apparatus is a recently developed and validated device for cardiorespiratory monitoring that allows for the simultaneous acquisition of these two signals [41,42,43,44]. In the IP method, a small electrical current below the tissue excitability threshold is applied through the application electrodes, and the voltage response is measured across the same or an additional pair of electrodes (receiving electrodes). As a person breathes, the air volume in the lungs changes, causing variations in the impedance within the chest, which are measured by the IP technique.

**Figure 1:**
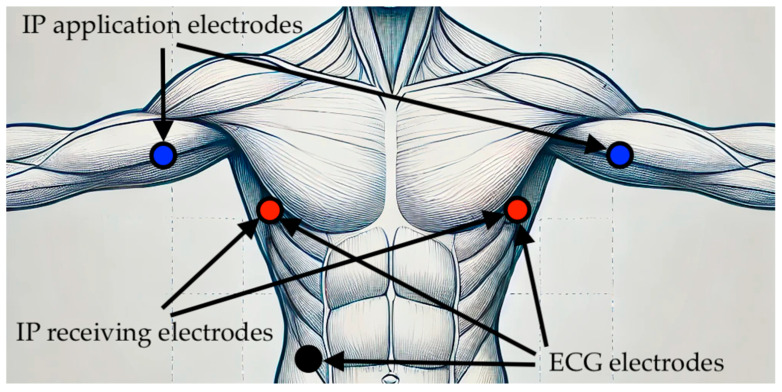
Placement of the electrodes used for the ECG and IP measurements.

A tetrapolar measurement using a sinusoidal current with an amplitude of up to 1 mA and a frequency of 100 kHz, along with electrode placement configured according to [45], was applied. Based on the findings in [46], it was presumed that such conditions allow for linear fitting to optimally align the IP with direct breathing measurements, e.g., using a facemask or nose cannula. Consequently, this alignment permits the IP signal to be treated as an equivalent to the relative TV. The placement of the electrodes used for the ECG and IP is presented in Figure 1.

In terms of ML, the modeling parameters derived from the cardiorespiratory recordings served as the model inputs and information about the group assignment was used as the output. This study was approved by two ethics committees (permissions: KB/55/N02/2019, 5 June 2019 and KB/70/2021, 14 June 2021) and conducted in accordance with the Declaration of Helsinki. Written informed consent forms were obtained from the legal guardians of subjects younger than 16 years old and directly from the subjects themselves if they were 16 years or older.

### 2.2. Signal Processing

**Figure 2:**
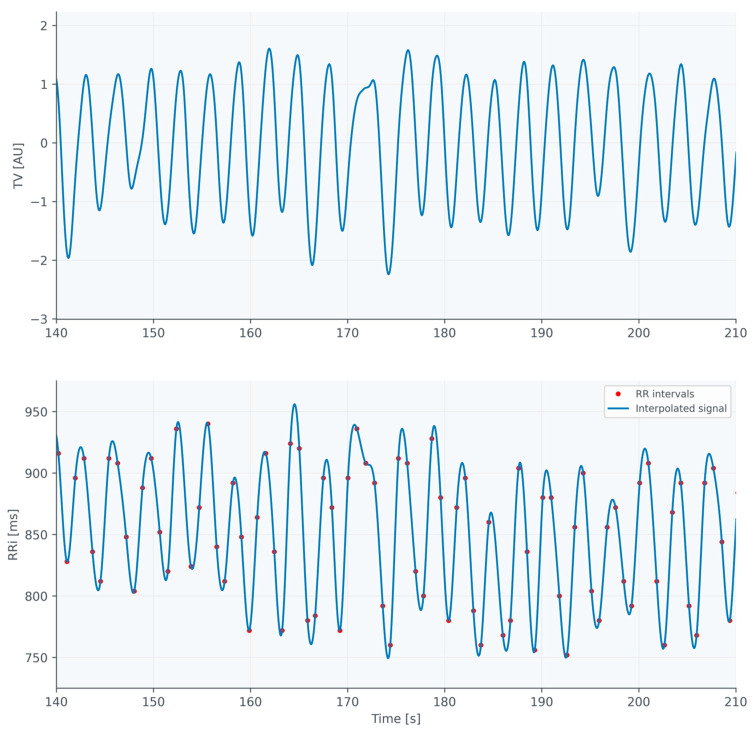
Examples of TV time series in the top chart and tachogram (blue line) with an individual RRi (red dots) in the bottom chart for Healthy subject #40.

Both the ECG and IP were acquired with a 250 Hz sampling frequency. The raw IP signal was filtered with a bandpass filter with cutoff frequencies of 0.05 and 0.67 Hz, corresponding to 3 and 40 breaths per minute, respectively; thus, the respiratory signal (Resp) was obtained. RR intervals (RRi) were extracted from the ECG signal using automatic detection, followed by manual correction by an experienced physician. The stationarity of the original RRi series was confirmed using the Phillips–Perron test. Such obtained series of RRi were interpolated using cubic interpolation in order to obtain a tachogram time series (RR) with the same sampling as the respiratory signal (which enabled estimating causal and information domain features based on the signals, not only beat-by-beat sequences). Both signals were then down-sampled to 25 Hz to reduce the computational complexity (only for the calculation of a subset of causal and information domain features). Examples of the obtained signals are presented in Figure 2.

### 2.3. Parameters Calculation

Three types of cardiorespiratory parameters were calculated: HRV (time and frequency domains and nonlinear), respiratory parameters, and parameters from causal and information domains. HRV parameters were calculated using the Neurokit2 package [47], extended with parameters from symbolic dynamics analysis [48]. From the respiratory signal, statistical characteristics, such as the RespRate, relative TV (indexed by the median TV due to the lack of calibration and the inability to convert the measured impedance signal directly into milliliters), and the inspiration/expiration time ratio, were derived. In terms of the causal relationships between cardiac and respiratory signals, features were calculated using the GC [49], the nonlincausality package with various ML models applied [18,50], the kernel GC [20], and the large-scale nonlinear Granger causality (lsNGC) [19]. Parameters for the information domain were mostly based on entropy analysis, but also simple statistics, like the highest Pearson correlation coefficient between the signals for a time lag between −1 and 1 s. The full list of features and their descriptions is presented in Appendix A, while the code used for their calculation is available in the repository [51]. As a result, for each patient, a total of 157 features were calculated, including 5 demographic (age, weight, height, sex, and body mass index), 102 cardiac, 18 respiratory, and 32 causal/information features.

### 2.4. Modeling

Based on the aforementioned parameters, four datasets (described further using the prefix D) utilized as input for machine learning modeling were created according to different types of features. Dataset D1 included demographic and cardiological features. Dataset D2 contained the same features as D1, with the addition of respiratory features. Dataset D3 expanded further by incorporating causal and information domain features. Finally, dataset D4 consisted of cardiological, respiratory, causal, and information domain features, excluding demographic data. The dataset components are presented in Table 1.

Moreover, two more datasets, D5 and D6, were created based on the 35 most influential features determined based on the Shapley values from datasets D3 and D4, respectively, in order to simplify the ML models, potentially further increase their accuracy, and evaluate the approach using features that most accurately reflected an individual’s health condition, making them preferable for future studies. Features for each patient were labeled according to their assigned group (Cardiac/Healthy/Sport). For the classification, various popular machine learning algorithms were utilized, including Logistic Regression (also with Ridge and Lasso regularization), Decision Tree, Support Vector Machine, Random Forest, Gradient Boosting, Naïve Bayes, K-Nearest Neighbors, AdaBoost, XGBoost, and multilayer perceptron. Hyperparameter optimization was applied for each algorithm. To validate the classification, 10-fold cross-validation was performed. In this method the dataset was randomly divided into 10 equal-sized subsets called folds. The ML model was trained on nine of these folds and tested on the remaining fold. This process was repeated 10 times, each time using a different fold as the test set and the remaining folds for training. The final model performance was then calculated as the average of the results from all 10 iterations, providing a more robust estimate of the model’s performance by reducing the variance associated with random sampling of the data into training and test sets.

The following metrics were calculated: accuracy, precision, recall, F1 score, Mathew’s correlation coefficient (MCC), and area under the curve (AUC) for each iteration on the test set according to Equations (4)–(8):

(4) $$Accuracy=\frac{1}{n}\sum_{i=1}^{n}1\left({\hat{y}}_{i}=y_{i}\right),$$

(5) $$Precision=\frac{TP}{TP+FP},$$

(6) $$Recall=\frac{TP}{TP+FN},$$

(7) $$F1score=\frac{T2*TP}{2*TP+FN+FP},$$

(8) $$MCC=\frac{n*\sum_{i=1}^{n}1\left({\hat{y}}_{i}=y_{i}\right)-\sum_{k}^{K}p_{k}*t_{k}}{\sqrt{\left(n^{2}-\sum_{k}^{K}p_{k}^{2}\right)*\left(n^{2}-\sum_{k}^{K}t_{k}^{2}\right)}}$$

where $1\left(x\right)$ is the indicator function, n is the number of samples, TP is true positive, FP is false positive, FN is false negative, $p_{k}$ is the number of times class k was predicted, and $t_{k}$ is the number of times class k truly occurred.

**Figure 3:**
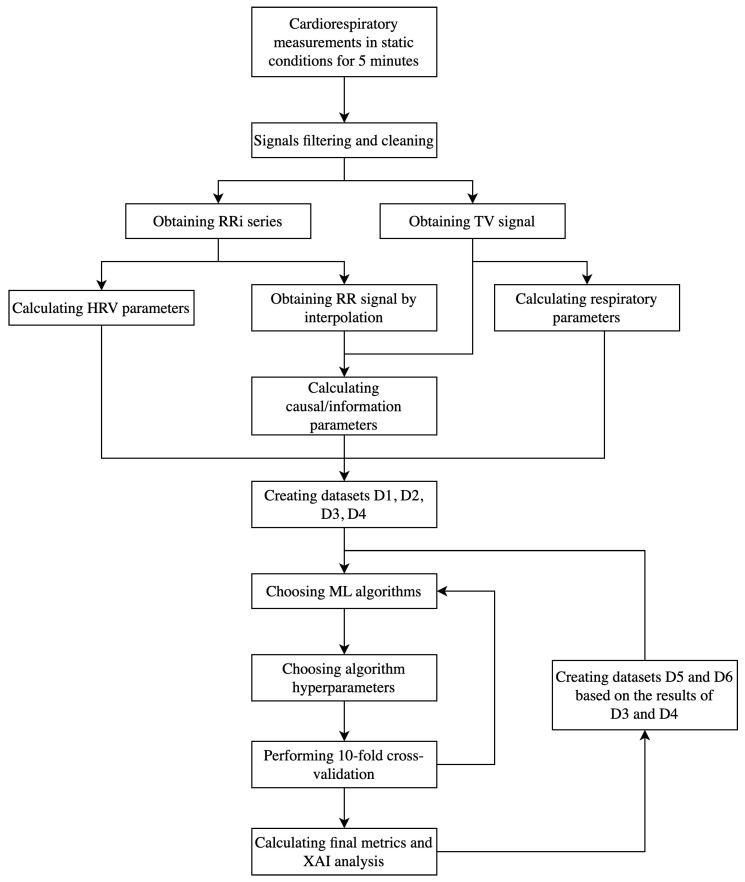
Diagram presenting the individual steps of the conducted analysis.

The mean values of the metrics from the cross-validation were treated as a final evaluation of the algorithm. The confusion matrix and receiver operating curve (ROC) were also visualized. In order to increase the training dataset and to handle class imbalance, upsampling using the synthetic minority oversampling technique (SMOTE) [52] was applied to the training set at each iteration of the validation. The code used for the modeling is presented in [51]. For each dataset, the best algorithm was determined based on the highest accuracy value, whose results were taken for further analysis. The metrics from individual iterations of cross-validation were compared between datasets using the pairwise Wilcoxon signed-rank test to determine whether the inclusion of certain feature types improved the classification performance. The assumed level of significance was 0.05. The analysis was performed using Python 3.10.8. A full diagram of the performed analysis is presented in Figure 3.

### 2.5. Explainable AI

To study the significance of the different features in the machine learning models, tools for XAI were utilized for the four datasets that obtained the best results in terms of accuracy. The Dalex Python package was used to assess which features were the most important for the model’s decisions using a permutation-based variable importance analysis [53]. Additionally, Shapley values were applied to understand how each feature influenced the individual predictions, which helped to explain the model’s behavior in more detail for individual subjects [54]. During each iteration of the cross-validation, the Shapley values and variable importance were determined based on 30 permutation rounds, using 1-AUC as the loss function for the test set. Following the complete cross-validation process, all the Shapley values for each data point and feature were collated and visualized, along with the average importance values of the variables.

## 3. Results

**Figure 4:**
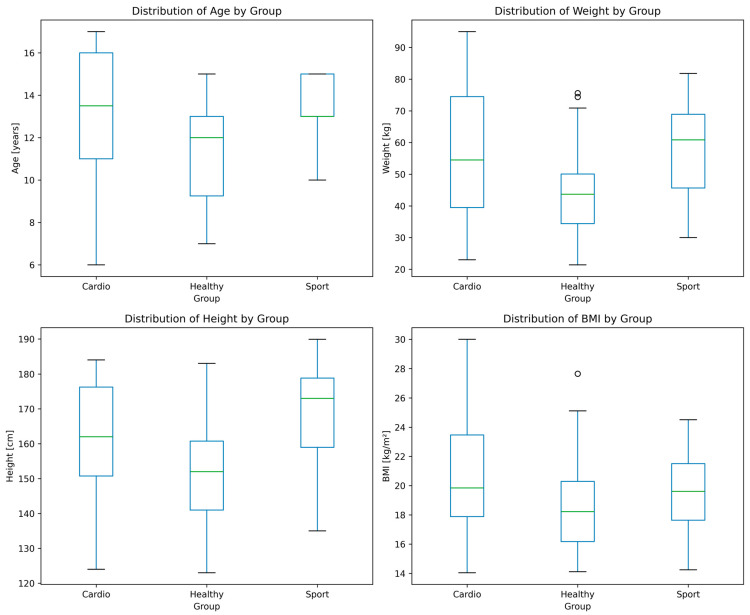
Distributions of the demographical parameters presented as boxplots. The central green line represents the median. Outliers, if present, are shown as individual points.

A total of 135 subjects (97 male and 38 female) were included in this study. The descriptive statistics of all groups are presented in Table 2. The Cardiac group consisted of patients with the following conditions: congenital heart defect (17), cardiomyopathy/myocarditis (8), and arrhythmia (7). The Sport group consisted of individuals with an average training experience of 5.82 ± 1.19 years (range 3–10 years) and a mean maximal oxygen uptake of 46.55 ± 4.42 mL/kg/min (range 39.4–57.9 mL/kg/min). The distributions of age, body mass, height, and body mass index (BMI) are presented in Figure 4. The demographic parameters of the participants were compared using the Kruskal–Wallis test, as the data did not follow a normal distribution. Although this test indicated statistically significant differences between the groups in terms of these parameters, they were widely overlapping. Assigning each individual subject to a given group based on any individual parameter was not possible; thus, advance machine learning modeling was utilized.

**Figure 5:**
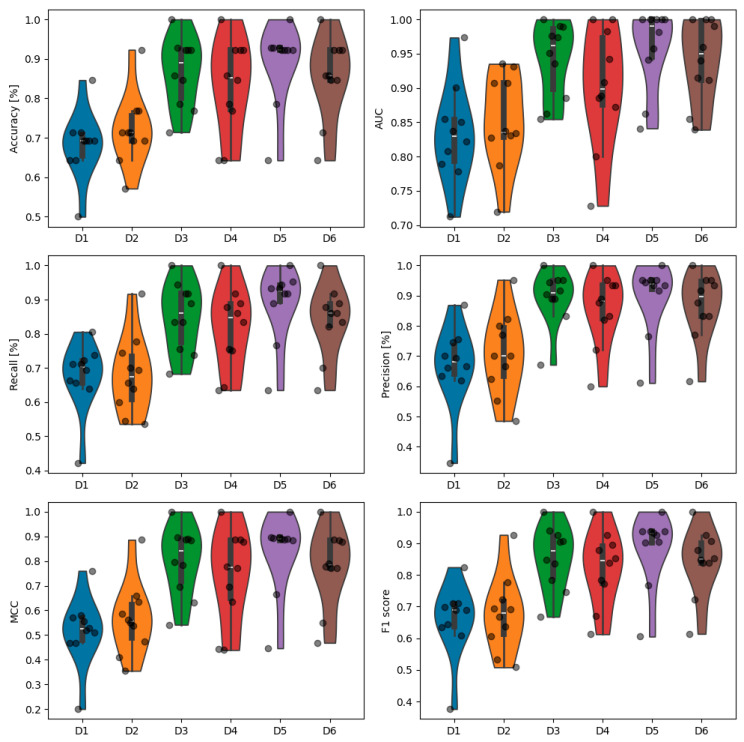
Violin plots of the metric values obtained from the cross-validation for each dataset. The metrics obtained from the individual iterations of 10-fold cross validation are presented as black dots.

**Figure 6:**
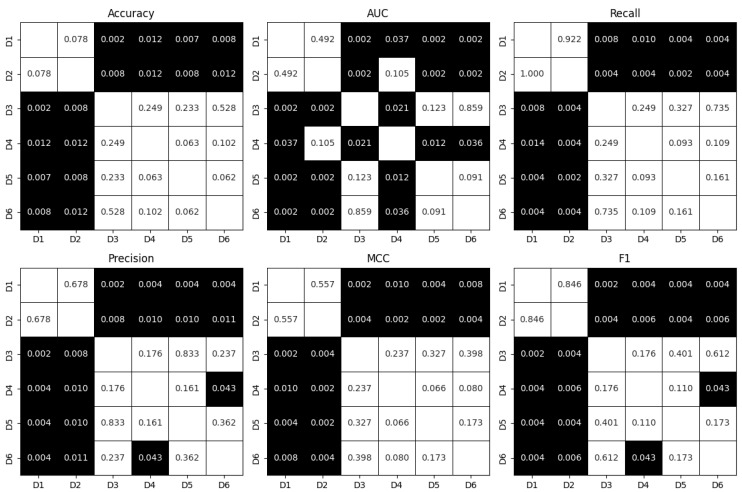
*p*-values from the Wilcoxon signed-rank test that compared the metrics obtained for individual datasets from individual iterations of 10-fold cross-validation. *p*-values smaller than 0.05, indicating statistically significant difference in the metric values, are highlighted with black backgrounds.

The metrics obtained for the best algorithm for each dataset alongside the upsampling proportions are presented in Table 3. The best results in terms of all metrics with accuracy equal to 89.1% were obtained for the fifth dataset, which incorporated demographic, cardiac, respiratory, causal, and information domain features while using the Gradient Boosting model. The selection of the most important features resulted in an improvement in the performance, as all the metrics for D5 and D6 were superior compared with the corresponding D3 and D4, respectively. Dataset D6, which did not leverage the demographic data, had an accuracy of 85.3% with the usage of the Gradient Boosting model. The violin plots of the metrics obtained during individual iterations of the 10-fold cross-validation are presented in Figure 5. Datasets D3 to D6 generally showed better performances across most metrics, with D5 typically demonstrating the best overall results. D1 and D2 had lower median values and wider distributions of metrics, indicating poorer and less consistent performance. The pairwise comparison of the obtain metrics between datasets using the Wilcoxon signed-rank test after cross-validation are presented in Figure 6. There was no statistical difference between the metrics for datasets D1 and D2, while all the other datasets had significantly better results than these two (despite the AUC for D4 compared with D2). Moreover, D4 had a significantly smaller AUC compared with D3, D5, and D6. There was also a significant difference in terms of the precision and F1 score between D4 and D6. The use of the limited datasets with the 35 most important features improved the performance, although not statistically significantly.

**Figure 7:**
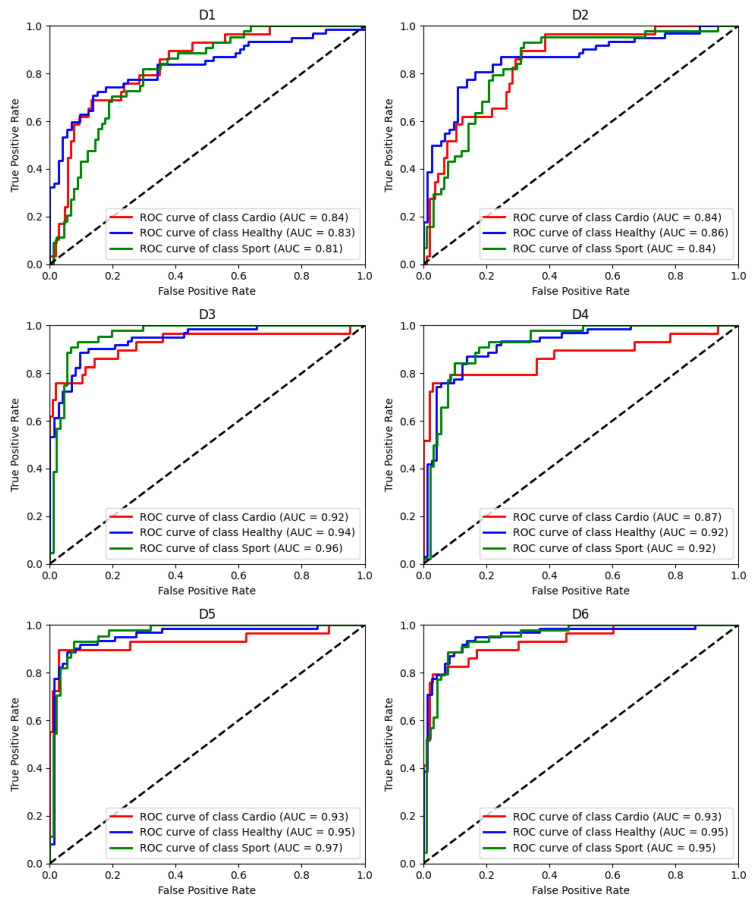
ROC and AUC values obtained for each considered dataset. The dashed black line represents the line of identity.

**Figure 8:**
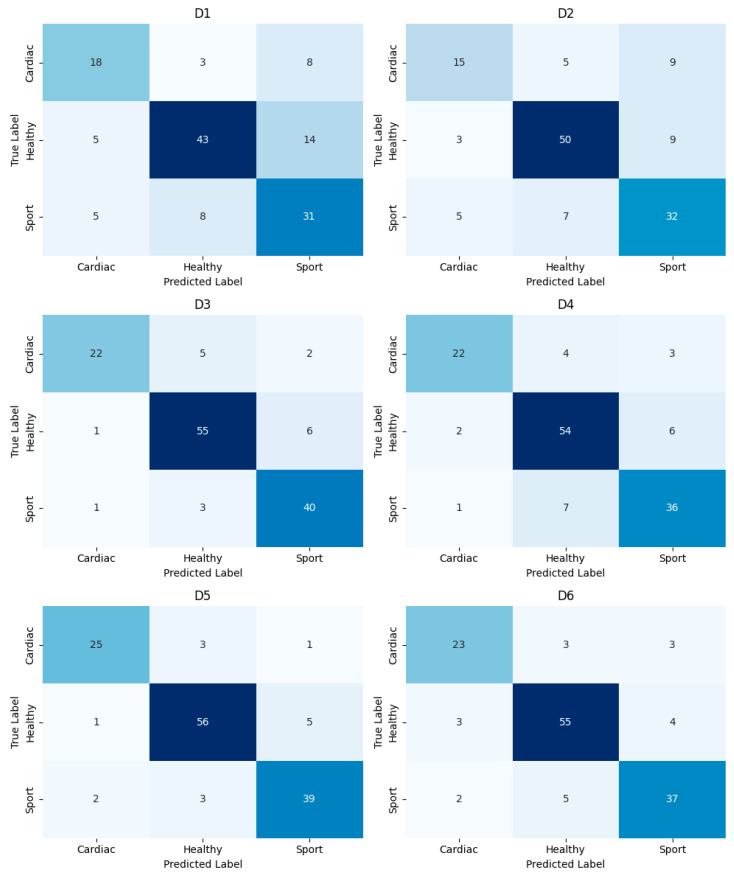
Cumulative confusion matrices obtained by summing the confusion matrices from the test set in each iteration of the 10-fold cross-validation for each considered dataset.

The ROC obtained on all predicted values on test sets are presented in Figure 7 for each group based on a one vs. all approach. The cumulative confusion matrices obtained for each dataset after the validation based on the test sets are presented in Figure 8.

**Figure 9:**
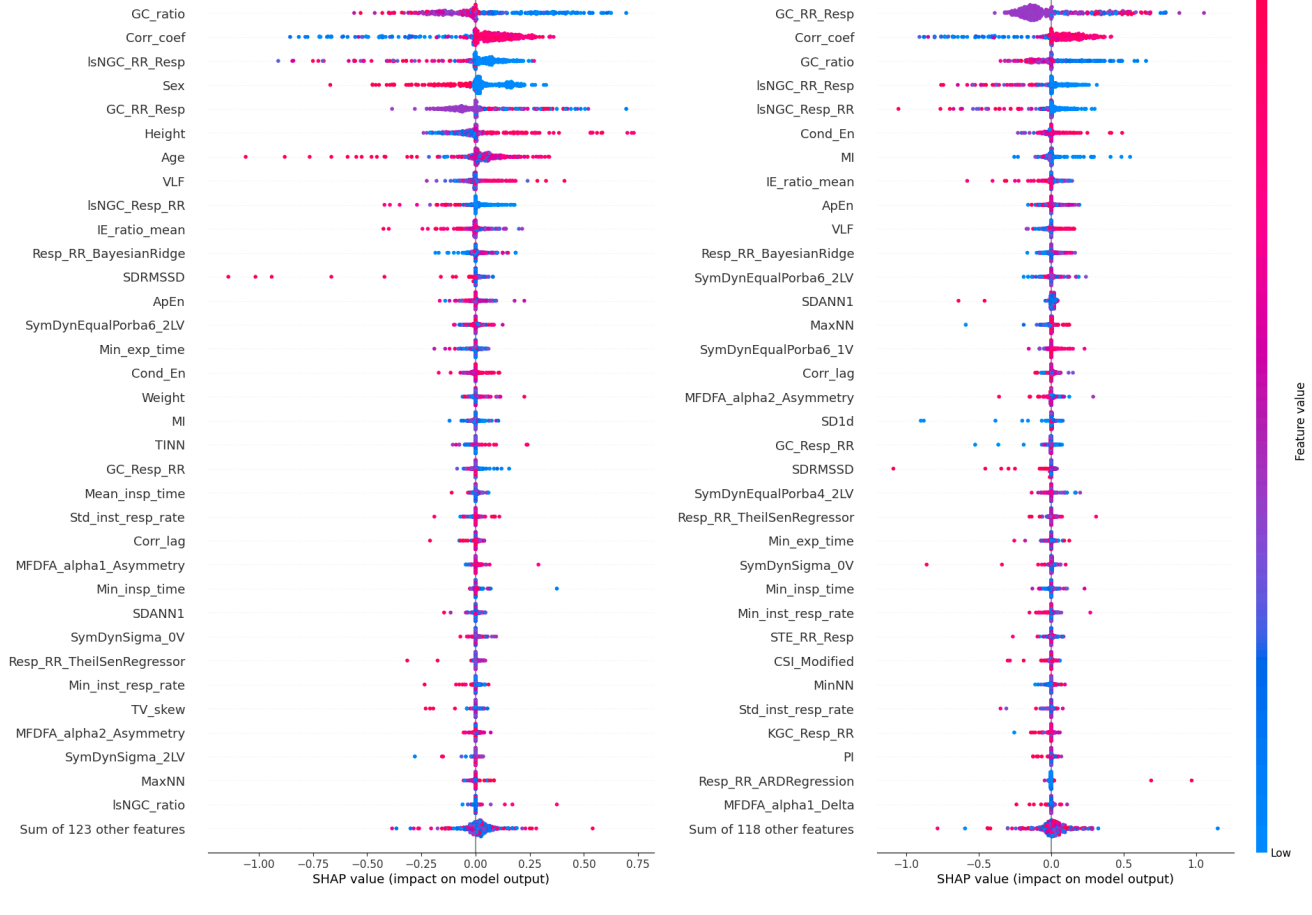
Shapley values obtained for the test data from the cross-validation for D3 (on the left) and D4 (on the right). The horizontal axis represents the SHAP value, which reflects the impact of each feature on the model’s output. The vertical axis lists the features in order of importance, with the most influential features at the top. The color of each dot represents the feature value for each data point: red dots correspond to high feature values, while blue dots correspond to low feature values.

**Figure 10:**
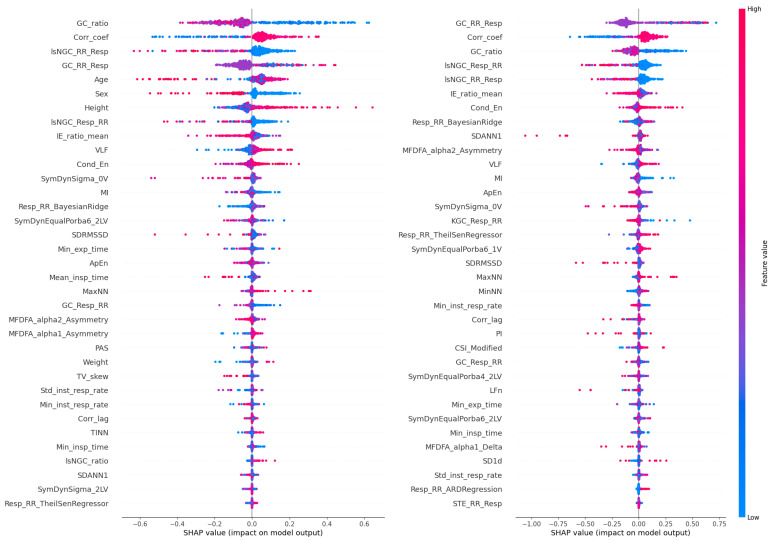
Shapley values obtained for the test data from the cross-validation for D5 (on the left) and D6 (on the right). The horizontal axis represents the SHAP value, which reflects the impact of each feature on the model’s output. The vertical axis lists the features in order of importance, with the most influential features at the top. The color of each dot represents the feature value for each data point: red dots correspond to high feature values, while blue dots correspond to low feature values.

**Figure 11:**
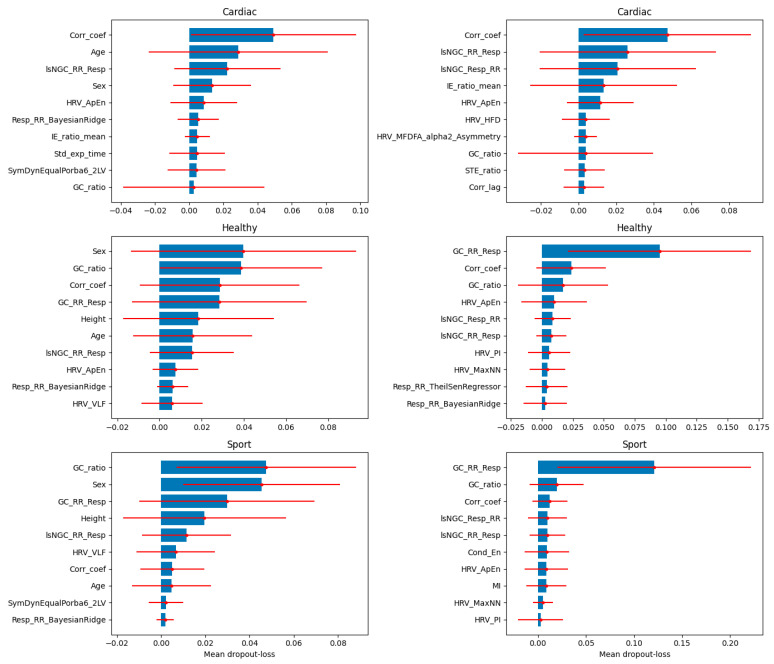
The mean values of dropout-loss variable importance are presented as bar plots with the standard deviation (red solid lines) for each class separately with a one vs. all approach applied for its calculations. The mean and standard deviation were calculated from the values of variable importance obtained at each iteration of the 10-fold cross-validation. The results for D3 are presented on the left and for D4 on the right.

**Figure 12:**
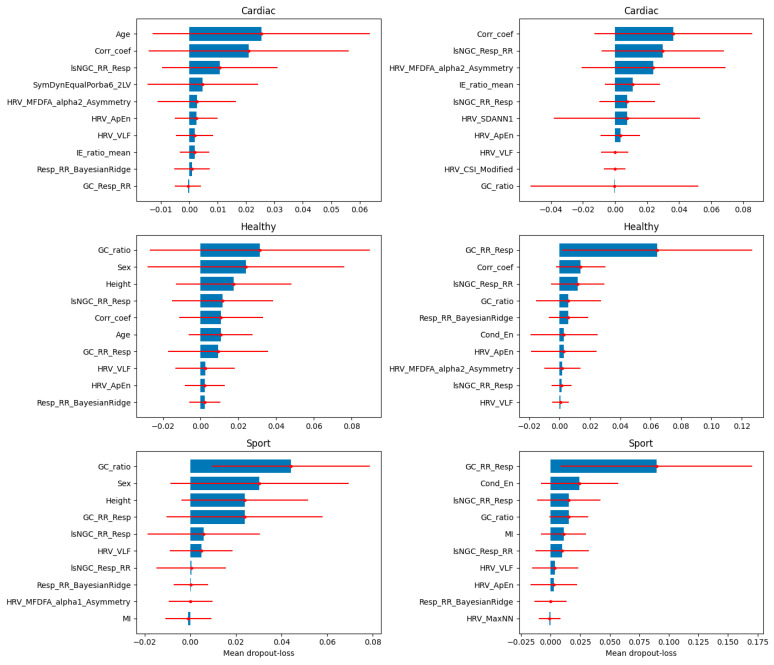
The mean values of the dropout-loss variable importance are presented as bar plots with the standard deviation (red solid lines) for each class separately with a one vs. all approach applied for its calculations. The mean and standard deviation were calculated from the values of variable importance obtained at each iteration of the 10-fold cross-validation. The results for D5 are presented on the left and for D6 on the right.

The results of the XAI analysis in terms of the Shapley values (presenting the contribution of each feature to the model’s predictions for individual samples) for datasets D3 and D4 (which contained all cardiorespiratory features) are presented in Figure 9, while D5 and D6 (which contained the most important features) are presented in Figure 10. Permutation-based variable importance (presenting the overall impact of each feature on the model’s performance) is visualized in Figure 11 for D3 and D4 and in Figure 12 for D5 and D6. For four analyzed datasets, some of the most influential features based on the Shapley values were as follows: the ratio of the GC from the respiratory signal to the tachogram (Resp→RR) by the GC from the tachogram to the respiratory signal (RR→Resp), the highest values of the Pearson correlation coefficient between the respiratory and cardiac signals for a lag between −1 and 1 s (CorrCoef), lsNGC RR→Resp, and GC RR→Resp. These features were also indicated as the most influential in the permutation-based variable importance analysis for distinguishing between the individuals from the Healthy and Sport groups (besides CorrCoef for dataset D5). In terms of distinguishing between the Cardiac and other groups, this analysis revealed that the CorrCoef and lsNGC RR→Resp features had the biggest impacts.

## 4. Discussion

We present the classification of young individuals into three distinct groups (Cardiac, Healthy, and Sport) based on cardiorespiratory parameters obtained from 5 min (rest, supine) measurements during spontaneous breathing using ML algorithms. The findings suggest that the integration of diverse cardiorespiratory parameters, including cardiac, respiratory, and causal/information domain features, significantly improved the accuracy and robustness of classification performance. Dataset D5, which incorporated the most influential parameters from all feature types, demonstrated superior performance across various metrics, including accuracy, recall, precision, AUC, MCC, and F1 score, as well as in terms of the shape of the ROC curves. The results obtained for D6 were similar in terms of most metrics, while it did not leverage the demographic information.

The high accuracy and other favorable metrics observed in the D5 dataset highlight the effectiveness of this approach in distinguishing between physiological profiles within classified groups. Moreover, in the case of misclassification, the Sport subjects were more often labeled as Healthy rather than Cardiac, and the Cardiac patients were more frequently mislabeled as Healthy rather than Sport subjects. This suggests a greater difference between the Cardiac and Sport groups in the feature space, with the Healthy group being somewhere in between, likely closer to the Sport group, as the Healthy subjects were mostly misclassified as Sport individuals. As also suggested in the previous work [55], the inclusion of causal and information domain features significantly improved the predictive models. The imperfect separation of the groups might have been due to changes in the cardiac and respiratory parameters that varied not only with the health status but also with age [56], which made it harder to distinguish the subjects between groups. Additionally, the heterogeneity of health issues in the Cardiac group could also negatively impact the accuracy, as different issues might be characterized by distinct cardiorespiratory profiles.

The observed improvement of classification for datasets containing causal and information features seems to support the initial hypothesis that cardiorespiratory interdependencies provide valuable diagnostic insights. This may be attributed to the additional information about the health status provided by the CRC, particularly the RSA phenomenon in which the change in the heart rate is caused by breathing with shortening of the RRi during the inhale and extension during the exhale [57]. Based on the HRV, information about the influence (in the causal sense) of respiration on the cardiac system might be obtained (primarily through frequency domain parameters) [58], although only taking into account the respiratory signal allowed for the full picture of the RSA to be captured. Existing literature seems to support the claim regarding the relevance of information related to CRC, as studies demonstrated that CRC plays an important role in sports medicine [10,59], e.g., allowing for differentiation between athletes and non-athletes [60], as an early marker of cardiac autonomic dysfunction in type 2 diabetes mellitus patients [61] and in research on obstructive sleep apnea [62,63].

The implementation of XAI tools confirmed that the inclusion of causal features was beneficial for the prediction accuracy, as some of them had a meaningful impact on the model output, both in terms of the Shapley values and permutation-based variable importance. Features related to RR→Resp causality had a bigger impact on the model than Resp→RR, which might seem contradictory to the RSA, which may be explained by the fact that the local maxima of the tachogram might occur before the local maxima of the respiratory signal [13,64], as well as physiological bidirectional character of interdependencies between the RR and TV signals [65]. This observation highlights the importance of interpreting causal and information domain features in the context of the underlying data and with respect to the domain knowledge. It is also noteworthy that, although the most influential causal domain features tended to be related to the traditional GC, nonlinear approaches, like lsNGC, were also among the most important parameters, indicating the complexity of the CRC phenomenon. The greater impact of linear features may be attributed to the static measurement conditions without introducing any interventions that could further emphasize the nonlinear relationships. It is also worth mentioning that despite the strong influence of demographic parameters on the model output and their statistical difference between the groups, dataset D6 provided satisfying results that reached over 85% accuracy based solely on features calculated from the cardiorespiratory signals without any information about the subjects’ demography. This allowed for the utilization of the method without the need for additional measurements of weight and height or knowledge about the subject’s age.

The utilization of ML algorithms with cardiorespiratory data in cardiology, pulmonology, and sports medicine has gained popularity in recent years [52,63,64,65,66]. The application of ML algorithms has been found useful in terms of coronary heart disease risk prediction [66], classifying exercise limitation severity [67], identifying integrative cardiopulmonary exercise test (CPET) profiles [68], the prediction of CRF in terms of the peak oxygen consumption [55], and central apnea detection in premature infants [69]. Despite the widespread application of ML in medicine, the integration of CRC-related features remains underexplored, with only a minority of studies incorporating these features [69]. In this study, we demonstrated that CRC-related features significantly improved the performance of the models, highlighting a gap in the literature and presenting a valuable opportunity for future research to further explore the role of CRC in various clinical and athletic contexts, as well as its impact on predictive modeling performance.

Moreover, the presented results demonstrate the potential of leveraging the ML-assisted evaluation of the health status based on static cardiorespiratory recordings. Such evaluation, which can be widely accessible due to the simplicity of the measurement process; the lack of need for advanced apparatus, like gas analyzers; and the absence of contraindications (as in the case of CPET [70]), is particularly valuable in areas such as pediatric heart transplantation [71], assessment of cardiovascular disease risk in adulthood [72], the monitoring of the cardiac rehabilitation progress [73], the timely identification of pathological conditions prior to sports events [74], and optimizing the training load and avoid overtraining [75]. Health status assessments are especially challenging in the pediatric population due to changes in cardiac and respiratory functions during maturation [76,77]. What is more, the interpretation of multiple cardiac, respiratory, and causal parameters might be challenging for the physician due to their multitude. Therefore, ML tools can simplify the data and provide an output in the form of a new, more interpretable parameter. The improvement in ML performance observed for datasets that contained only the 35 most important features, compared with the corresponding datasets with all cardiorespiratory features, although not statistically significant, highlighted the need for research into identifying the optimal parameter set that would provide the highest diagnostic value.

Models developed in this study, although of the general purpose, could be potentially useful for initial patient screening. Foremost, they could be further personalized and specialized, e.g., based on systematically conducted measurements during training camps or rehabilitation processes with the training/rehabilitation outcome as model targets. After further development for the specific use case, the presented method, integrating various easily accessible cardiorespiratory features and machine learning, would be especially helpful in clinical practice by providing more personalized and precise health assessments. Specifically, it could aid in cardiac rehabilitation by offering a non-invasive, monitoring solution that leverages not only the typically used cardiological parameters (like linear HRV ones), but a broad range of cardiorespiratory features, including nonlinear CRC parameters and machine learning models to track patient progress through the rehabilitation process. The method’s ability to classify individuals based on their cardiorespiratory signals could also improve the early detection of potential health issues, enabling timely interventions and more tailored rehabilitation strategies.

Additionally, its application could extend to optimizing training loads in athletes. The ML-assisted parametrization of cardiorespiratory data based on the presented approach would allow coaches and sports physicians to closely monitor athletes’ adaptation to training, ensuring they do not exceed their physiological limits and reducing the risk of overtraining or injury. In broader healthcare contexts, this method could be applied to monitor post-operative recovery, where the continuous, non-invasive tracking of cardiorespiratory functions could help detect complications early, such as signs of respiratory distress or cardiovascular instability. However, further studies and model training are needed to optimize the method’s predictive power and ensure its accuracy and reliability in those clinical applications.

The limitation of this study was the absence of female subjects in the Sport group, as well as variations in the group sample sizes and demographic parameters, along with the heterogeneity of health issues in the Cardiac group, all of which might have negatively impacted the performance of the ML models. Including patients with arrhythmias could also be seen as a potential limitation. These patients may experience paroxysmal arrhythmias, and the cardiorespiratory parameters measured outside of an arrhythmia episode might not differ significantly from those of healthy subjects. However, the condition itself could indirectly impact the cardiorespiratory profile through lifestyle changes, such as avoiding physical exercise. A larger sample size with an equal distribution of demographic parameters and increased within-group homogeneity would be beneficial from the perspective of training the machine learning models. Moreover, the fact that subjects in the Sport group only practiced a single sport discipline could also be considered a limitation.

As a result of this study, we not only trained classification models for multiple health conditions that may be useful for initial patient screening but also highlighted the significance of causal and information domain parameters related to CRC and identified a subset of cardiorespiratory features that could be further explored. Our study demonstrated that expanding the most commonly used HRV parameters with respiratory and CRC data could lead to improved subject profiling. These findings have the potential to be leveraged in predictive modeling to monitor parameter trends in individual progress during training or rehabilitation, as well as in the context of CRF and specific cardiac conditions. However, additional research is necessary to further explore these applications.

## 5. Conclusions

This study demonstrated the utilization of ML algorithms with a wide variety of cardiorespiratory features in the classification of pediatric individuals into three groups based on their health statuses while identifying the optimal set of cardiorespiratory features with potential for further use in personalized medical modeling. The results also emphasize the value of including causal and information domain features in the assessment of individuals’ health statuses, as these features allowed for significant improvement of the classification accuracy.

## Figures and Tables

**Table 1:** Description of the content of each dataset based on the type of features, where “+” indicates that the given features are included in the respective dataset.

Dataset	Demographic Data	Cardiological Features	Respiratory Features	Causal and Information Domain Features
D1	+	+		
D2	+	+	+	
D3	+	+	+	+
D4		+	+	+

**Table 2:** Descriptive statistics of all three study groups and the overall study population. Values are presented as the mean ± standard deviation and the range of the parameter in brackets.

	Cardiac	Healthy	Sport	Overall
N	29	62	44	135
Male/female	20/9	33/29	44/0	97/38
Age	13.1 ± 3.5 (6–17)	11.0 ± 2.2 (7–15)	13.3 ± 1.4 (10–15)	12.2 ± 2.6 (6–17)
Body mass [kg]	57.1 ± 21.0 (23.0–95.0)	43.5 ± 12.1 (21.4–75.6)	57.2 ± 13.6 (30.0–81.8)	50.9 ± 16.4 (21.4–95.0)
Height [cm]	160.4 ± 17.2 (123–184)	151.2 ± 13.1 (123–183)	169.4 ± 12.7 (135–190)	159.1 ± 16.0 (123–190)
HR [beats/min]	72.8 ± 13.3 (56.0–100.5)	79.4 ± 10.2 (60.7–100.5)	76.9 ± 15.0 (46.7–121.4)	77.2 ± 12.8 (46.7–121.4)
RMSSD [ms]	55.3 ± 36.8 (9.4–140.7)	61.8 ± 34.4 (13.0–162.3)	68.2 ± 46.7 (5.6–178.9)	62.5 ± 39.6 (5.6–178.9)
RespRate [breaths/min]	18.5 ± 4.6 (7.9–25.4)	18.8 ± 3.5 (10.7–28.5)	17.1 ± 3.5 (10.2–25.8)	18.2 ± 3.8 (7.9–28.5)

**Table 3:** Mean ± standard deviation of metrics obtained from the 10-fold cross-validation for the given ML algorithm with the applied SMOTE upsampling technique with the strategy presented as a number of Cardiac/Healthy/Sport training samples.

	D1	D2	D3	D4	D5	D6
Accuracy [%]	68.3 ± 8.1	72.0 ± 8.7	86.7 ± 8.4	83.1 ± 11.5	89.1 ± 9.6	85.3 ± 10.0
AUC	83.2 ± 6.7	85.2 ± 6.5	94.2 ± 5.2	90.1 ± 8.3	95.8 ± 5.7	94.1 ± 5.7
Recall [%]	67.6 ± 9.6	68.1 ± 10.9	85.1 ± 9.6	81.6 ± 11.2	88.9 ± 10.2	84.0 ± 9.9
Precision [%]	66.9 ± 12.7	70.8 ± 13.0	89.5 ± 8.6	85.6 ± 11.3	89.6 ± 11.1	86.9 ± 10.6
MCC	0.516 ± 0.132	0.566 ± 0.140	0.801 ± 0.133	0.742 ± 0.180	0.835 ± 0.151	0.778 ± 0.152
F1 score	0.659 ± 0.109	0.676 ± 0.114	0.856 ± 0.095	0.823 ± 0.111	0.885 ± 0.109	0.843 ± 0.102
ML algorithm	XGBoost Classifier	Logistic Regression	Gradient Boosting	Gradient Boosting	Gradient Boosting	Gradient Boosting
Upsampling strategy	200/200/200	200/200/150	200/200/200	200/200/200	200/200/200	200/200/200

## Data Availability

Data and materials used in this study are available upon reasonable request to the corresponding author and under a collaboration agreement.

## Abbreviations

The following abbreviations are used in this manuscript:

AUC	Area under the curve
BMI	Body mass index
CRC	Cardiorespiratory coupling
CRF	Cardiorespiratory fitness
CPET	Cardiopulmonary exercise test
ECG	Electrocardiography
GC	Granger causality
HRV	Heart rate variability
IP	Impedance pneumography
lsNGC	Large-scale nonlinear Granger causality
MCC	Mathew’s correlation coefficient
ML	Machine learning
Resp	Respiratory signal
RespRate	Respiratory rate
ROC	Receiver operating curve
RR	Tachogram time series
RRi	RR intervals
RSA	Respiratory sinus arrhythmia
SMOTE	Synthetic minority oversampling technique
TV	Tidal volume
XAI	Explainable artificial intelligence

## Appendix A

Full list of features used in this study and their descriptions [18,19,20,47,48,49,50,78,79,80,81].

**Demography**

- Sex
- Age
- Weight
- Height
- BMI: Body mass index

**Cardiac**

- MeanNN: The mean of the RR intervals.
- SDNN: The standard deviation of the RR intervals.
- SDANN1: The standard deviation of average RR intervals extracted from 1-min segments of time series data.
- SDNNI1: The mean of the standard deviations of RR intervals extracted from 1-min segments of time series data.
- RMSSD: The square root of the mean of the squared successive differences between adjacent RR intervals.
- SDSD: The standard deviation of the successive differences between RR intervals.
- CVNN: The standard deviation of the RR intervals (SDNN) divided by the mean of the RR intervals (MeanNN).
- CVSD: The root mean square of successive differences (RMSSD) divided by the mean of the RR intervals (MeanNN).
- MedianNN: The median of the RR intervals.
- MadNN: The median absolute deviation of the RR intervals.
- MCVNN: The median absolute deviation of the RR intervals (MadNN) divided by the median of the RR intervals (MedianNN).
- IQRNN: The interquartile range (IQR) of the RR intervals.
- SDRMSSD: SDNN/RMSSD, a time-domain equivalent for the low Frequency-to-High Frequency (LF/HF) Ratio.
- Prc20NN: The 20th percentile of the RR intervals.
- Prc80NN: The 80th percentile of the RR intervals.
- pNN50: The proportion of RR intervals greater than 50 ms, out of the total number of RR intervals.
- pNN20: The proportion of RR intervals greater than 20 ms, out of the total number of RR intervals.
- MinNN: The minimum of the RR intervals.
- MaxNN: The maximum of the RR intervals.
- HTI: The HRV triangular index, measuring the total number of RR intervals divided by the height of the RR intervals histogram.
- TINN: The baseline width of the RR intervals distribution obtained by triangular interpolation.
- VLF: The spectral power of very low frequencies (0.0033 to 0.04 Hz).
- LF: The spectral power of low frequencies (0.04 to 0.15 Hz).
- HF: The spectral power of high frequencies (0.15 to 0.4 Hz).
- VHF: The spectral power of very high frequencies (0.4 to 0.5 Hz).
- TP: The total spectral power.
- LFHF: The ratio obtained by dividing the low frequency power by the high frequency power.
- LFn: The normalized low frequency, obtained by dividing the low frequency power by the total power.
- HFn: The normalized high frequency, obtained by dividing the low frequency power by the total power.
- LnHF: The log transformed HF.
- SD1: Standard deviation perpendicular to the line of identity.
- SD2: Standard deviation along the identity line. Index of long-term HRV changes.
- SD1SD2: ratio of SD1 to SD2.
- S: Area of ellipse described by SD1 and SD2 (pi * SD1 * SD2).
- CSI: The Cardiac Sympathetic Index calculated by dividing the longitudinal variability of the Poincaré plot (4*SD2) by its transverse variability (4*SD1).
- CVI: The Cardiac Vagal Index equal to the logarithm of the product of longitudinal (4*SD2) and transverse variability (4*SD1).
- CSI_Modified: The modified CSI obtained by dividing the square of the longitudinal variability by its transverse variability.
- GI: Guzik’s Index.
- SI: Slope Index.
- AI: Area Index.
- PI: Porta’s Index.
- SD1d and SD1a: short-term variance of contributions of decelerations (prolongations of RR intervals) and accelerations (shortenings of RR intervals), respectively.
- C1d and C1a: the contributions of heart rate decelerations and accelerations to short-term HRV, respectively.
- SD2d and SD2a: long-term variance of contributions of decelerations (prolongations of RR intervals) and accelerations (shortenings of RR intervals), respectively.
- C2d and C2a: the contributions of heart rate decelerations and accelerations to long-term HRV, respectively.
- SDNNd and SDNNa: total variance of contributions of decelerations (prolongations of RR intervals) and accelerations (shortenings of RR intervals), respectively.
- Cd and Ca: the total contributions of heart rate decelerations and accelerations to HRV.
- PIP: Percentage of inflection points of the RR intervals series.
- IALS: Inverse of the average length of the acceleration/deceleration segments.
- PSS: Percentage of short segments.
- PAS: Percentage of NN intervals in alternation segments.
- DFA_alpha1: The monofractal detrended fluctuation analysis of the HR signal, corresponding to short-term correlations.
- DFA_alpha2: The monofractal detrended fluctuation analysis of the HR signal, corresponding to long-term correlations.
- MFDFA_alpha1_Width, MFDFA_alpha1_Peak, MFDFA_alpha1_Mean, MFDFA_alpha1_Max, MFDFA_alpha1_Delta, MFDFA_alpha1_Asymmetry, MFDFA_alpha1_Fluctuation, MFDFA_alpha1_Increment, MFDFA_alpha2_Width, MFDFA_alpha2_Peak, MFDFA_alpha2_Mean, MFDFA_alpha2_Max, MFDFA_alpha2_Delta, MFDFA_alpha2_Asymmetry, MFDFA_alpha2_Fluctuation, MFDFA_alpha2_Increment: Indices related to the Multifractal Detrended Fluctuation Analysis.
- ApEn: Approximate entropy.
- SampEn: Sample entropy.
- ShanEn: Shannon entropy.
- FuzzyEn: Fuzzy entropy.
- MSEn: Multiscale entropy.
- CMSEn: Composite Multiscale entropy.
- RCMSEn: Refined Composite Multiscale entropy.
- CD: Correlation Dimension.
- HFD: Higuchi’s Fractal Dimension.
- KFD: Katz’s Fractal Dimension.
- LZC: Lempel-Ziv Complexity.
- SymDynMaxMin_0V: Percentage of words in the Max–min method that fall into the 0V family, representing sequences where all three consecutive symbols are equal. This method uses six levels of uniform quantization.
- SymDynMaxMin_1V: Percentage of words in the Max–min method that fall into the 1V family, which includes sequences with only one variation among three consecutive symbols.
- SymDynMaxMin_2LV: Percentage of words in the Max–min method that fall into the 2LV family, representing sequences with two variations in the same direction, forming an increasing or decreasing sequence.
- SymDynMaxMin_2UV: Percentage of words in the Max–min method that fall into the 2UV family, where symbols vary two times in opposite directions, forming a peak or a valley.
- SymDynSigma_0V: Percentage of words in the σ method that fall into the 0V family. The σ method uses three levels defined by the signal average and its variations shifted up and down by a set factor.
- SymDynSigma_1V: Percentage of words in the σ method that fall into the 1V family.
- SymDynSigma_2LV: Percentage of words in the σ method that fall into the 2LV family.
- SymDynSigma_2UV: Percentage of words in the σ method that fall into the 2UV family.
- SymDynEqualPorba4_0V: Percentage of words using the Equal-probability method with four quantization levels (q = 4) that fall into the 0V family.
- SymDynEqualPorba4_1V: Percentage of words using the Equal-probability method with four quantization levels that fall into the 1V family.
- SymDynEqualPorba4_2LV: Percentage of words using the Equal-probability method with four quantization levels that fall into the 2LV family.
- SymDynEqualPorba4_2UV: Percentage of words using the Equal-probability method with four quantization levels that fall into the 2UV family.
- SymDynEqualPorba6_0V: Percentage of words using the Equal-probability method with six quantization levels (q = 6) that fall into the 0V family.
- SymDynEqualPorba6_1V: Percentage of words using the Equal-probability method with six quantization levels that fall into the 1V family.
- SymDynEqualPorba6_2LV: Percentage of words using the Equal-probability method with six quantization levels that fall into the 2LV family.
- SymDynEqualPorba6_2UV: Percentage of words using the Equal-probability method with six quantization levels that fall into the 2UV family.

**Respiratory**

- RespRate: respiratory rate.
- Std_inst_resp_rate: Standard deviation of instantaneous respiratory rate.
- Min_inst_resp_rate: minimal value of instantaneous respiratory rate.
- Max_inst_resp_rate: maximal value of instantaneous respiratory rate.
- Mean_insp_time: mean inspiration time.
- Min_insp_time: minimal inspiration time.
- Max_insp_time: maximal inspiration time.
- Std_insp_time: standard deviation of inspiration time.
- Mean_exp_time:mean expiration time.
- Min_exp_time: minimal expiration time.
- Max_exp_time: maximal expiration time.
- Std_exp_time: standard deviation of expiration time.
- TV_std: standard deviation of tidal volume normalized by median tidal volume.
- TV_q25: 25th quantile of tidal volume normalized by median tidal volume.
- TV_q75: 75th quantile of tidal volume normalized by median tidal volume.
- TV_skew: skewness of tidal volume normalized by median tidal volume.
- TV_kurtosis: kurtosis of tidal volume normalized by median tidal volume.
- IE_ratio_mean: mean inspiration/expiration ratio.

**Causal/Information**

- GC_RR_Resp: Granger causality from tachogram to respiratory signal.
- GC_Resp_RR: Granger causality from respiratory signal to tachogram.
- STE_RR_Resp: Symbolic transfer entropy from tachogram to respiratory signal.
- STE_Resp_RR: Symbolic transfer entropy from respiratory signal to tachogram.
- Resp_RR_SVR: Granger causality from respiratory signal to tachogram calculated using Support Vector Regression (SVR).
- RR_Resp_SVR: Granger causality from tachogram to respiratory signal calculated using Support Vector Regression (SVR).
- Resp_RR_BayesianRidge: Granger causality from respiratory signal to tachogram calculated using Bayesian Ridge Regression.
- KGC_Resp_RR: Granger causality from respiratory signal to tachogram calculated using Kernel Granger Causality (KGC).
- KGC_RR_Resp: Granger causality from Tachogram to respiratory signal calculated using Kernel Granger Causality (KGC).
- RR_Resp_GradientBoostingRegressor: Granger causality from tachogram to respiratory signal calculated using Gradient Boosting Regressor.
- Resp_RR_GradientBoostingRegressor: Granger causality from respiratory signal to tachogram calculated using Gradient Boosting Regressor.
- RR_Resp_TheilSenRegressor: Granger causality from tachogram to respiratory signal calculated using Theil-Sen Regressor.
- Resp_RR_TheilSenRegressor: Granger causality from respiratory signal to tachogram calculated using Theil-Sen Regressor.
- RR_Resp_ARDRegression: Granger causality from tachogram to respiratory signal calculated using Automatic Relevance Determination (ARD) Regression.
- Resp_RR_ARDRegression: Granger causality from respiratory signal to tachogram calculated using Automatic Relevance Determination (ARD) Regression.
- RR_Resp_RandomForestRegressor: Granger causality from tachogram to respiratory signal calculated using Random Forest Regression.
- Resp_RR_RandomForestRegressor: Granger causality from respiratory signal to tachogram calculated using Random Forest Regression.
- lsNGC_RR_Resp: Large scale-nonlinear Granger causality from tachogram to respiratory signal.
- lsNGC_Resp_RR: Large scale-nonlinear Granger causality from respiratory signal to tachogram.
- Corr_coef: Highest values of the Pearson correlation coefficient between respiratory and cardiac signals for lag between −1 and 1 s.
- Corr_lag: Value of the lag for which the highest Pearson correlation coefficient was obtained.
- MI: Mutual information.
- AI: Active information.
- Block_En: Block entropy.
- Cond_En: Conditional entropy.
- En_rate: Entropy rate.
- Trans_En: Transfer entropy
- Perm_En: Permutation entropy.
- KGC_ratio: ratio of KGC_Resp_RR and KGC_RR_Resp.
- GC_ratio: ratio of GC_Resp_RR and GC_RR_Resp.
- STE_ratiols: ratio of STE_Resp_RR and STE_RR_Resp.
- lsNGC_ratio: ratio of lsNGC_Resp_RR and lsNGC_RR_Resp.
